# Cerebral ambiguity

**DOI:** 10.1002/brb3.277

**Published:** 2014-09-17

**Authors:** Claudio Baracchini

**Affiliations:** Department of Neurological Sciences, University of Padua School of MedicineVia N. Giustiniani 5, Padova, 35128, Italy

Please note that an article related to this editorial, “Factors affecting the determination of cerebrovascular reactivity,” doi: 10.1002/brb3.275, can be found here, also published in *Brain and Behavior*.

## Factors affecting cerebrovascular reactivity measurement

What do we really know about cerebral blood flow (CBF) control? Within the boundaries of our current knowledge, is there an easy test of clinical importance that can eloquently describe the status quo of CBF regulatory processes?

Remarkably, the brain alone consumes 20% of the body's chemical energy, even though it accounts for only 2% of the body's mass. This high consumption of energy is crucial for the normal functioning of the brain, which needs to be constantly perfused as changes in perfusion lead to an immediate alteration in brain function. If blood glucose and oxygen are not adequately supplied to a region of the brain as in ischemic stroke or cerebral palsy, neurons, and glia become impaired or die.

To sustain neuronal function, the brain has evolved very complex regulatory processes to ensure a continuous and constant blood supply (Attwell et al. 2010; Peterson et al. 2011; Willie et al. 2014). The first mechanism is cerebral pressure autoregulation, a process whereby the cerebral arterioles maintain a constant flow despite changes of cerebral perfusion pressure. The second is neurovascular coupling which refers to the brain's ability to increase the flow of blood to regions where neurons are metabolically active, a response also termed functional hyperemia. Metabolic messengers such as adenosine and lactate and chemical stimuli like carbon dioxide (CO_2_) contribute to functional hyperemia through glutamate-induced prostaglandin signaling to blood vessels. The effect is a dilatation of the arterioles which leads to a CBF increase; recent data have also suggested the important role of pericytes in regulating CBF through the control of capillary diameter (Itoh and Suzuki 2012). The third mechanism is the neurogenic regulation whereby extensive arborization of perivascular nerves play a role in controlling CBF. Essential to all three regulatory processes is the neurovascular unit, composed of endothelial cells, perivascular nerves, and astrocytes; the endothelium acts through several vasoactive factors (nitric oxide, endothelium-dependent hyperpolarization factor, eicosanoids, endothelins), while astrocytic foot processes directly abut the blood vessels and by glutamate-mediated signaling also play a key role in the regulation of CBF. The interaction of these complex regulatory mechanisms, as well as the mechanisms themselves, are not fully understood. However, their importance is highlighted by the fact that significant brain injury occurs when these regulatory mechanisms are lost as in hypertension, diabetes, stroke, Alzheimer's disease, spinal cord injury. This underscores the need of research in this field, the results of which might have important implications for the development of new therapeutic approaches.

In this issue of *Brain and Behavior*, Regan and colleagues present a very interesting ultrasonographic study on nine young and healthy subjects, with the aim of elucidating the factors that affect cerebrovascular reactivity (CVR) measurement. In particular they investigated how changes in mean arterial pressure (MAP), different body positions (sitting vs. supine), different stimulus patterns (step vs. ramp) and analysis techniques affect the calculation of CVR. They used CO_2_ as the stimulus and transcranial Doppler (TCD) measurement of middle cerebral artery velocity (MCAv) as the response. They found that MAP increases with CO_2_ thus acting as a confounding factor for CVR measurement; hence, they suggest that blood pressure should be monitored during CVR testing. Furthermore, CVR seems to depend also on the stimulus pattern used: lower CVR values were obtained for step response compared to ramp response. Finally, they observed that CVR is not affected by subject's position (sitting vs. supine). The authors conclude that when testing for CVR, MAP, and stimulus pattern should be taken into account before interpreting the results.

Cerebrovascular reactivity is the ability of the peripheral resistance vessels (arterioles and small pial vessels) to dilate and constrict to vasoactive stimuli such as CO_2_ (Huber and Handa 1967), and this response can be easily assessed by TCD using blood flow velocity as a surrogate for CBF (Ringelstein et al. 1992).

Consequently, CVR testing has become a widely accepted and useful tool to stratify risk in patients with acute cerebral ischemia (Palazzo et al. 2010) or with carotid artery obstruction (Silvestrini et al. 2000), and to monitor treatment efficacy (Baracchini et al. 2006). In light of this, it was surprising to see that none of the study subjects, regardless of the small number, underwent an examination of the cervical vessels to exclude the presence of carotid steno-occlusive disease. In fact, patients with a > 50% stenosis of the common carotid artery or internal carotid artery or MCA, as detected by extracranial or transcranial ultrasound, should be excluded in order to avoid misinterpretation of findings.

Emphasis should also be given to another issue: comparison between CVR assessment and cerebral autoregulation (CA) testing. In CVR assessment, a vasodilatatory stimulus (breath-holding, acetazolamide injection, dose-controlled inhalation of CO_2_) is applied with the patient in the supine position for a time sufficient to induce the smooth muscle response and modify CBF. Using TCD the increase in CBF can be shown by measuring the mean blood flow velocity (MFV) in the main stem of the MCA. Assuming that the diameter of the MCA is constant and vasodilatation occurs only in the periphery, MFV is proportional to CBF. CVR is expressed as the percentage increase in MFV after the vasodilatatory stimulus is applied and adjusted for the time it takes for the response to develop: a significant increase in MFV represents a preserved CVR, no MFV increase means an exhausted CVR and a decrease in MFV stands for a hemodynamic steal.

Cerebral autoregulation is defined as the capability of the cerebral blood vessels to maintain constant CBF despite the changes in MAP and according to the metabolic need of the brain (Aaslid et al. 1989). This ensures that perfusion of any population of neurons is kept independent from systemic hemodynamic oscillations over a wide range. The mechanisms of CA are still poorly understood, but are undoubtedly multifactorial; in fact, CA can be affected by a number of physiological parameters, such as transmural pressure, CO_2_, autonomic function, intracranial pressure, etc., whereas CVR is solely in connection with vasodilation or vasoconstriction. Therefore, when assessing CA several vasomotor mechanisms are tested to see if CBF levels are maintained at different blood pressure values; currently, however, there is no uniform technique for investigating CA.

In accordance with other authors (Dumville et al. 1998), Regan et al. observed that in CO_2_ reactivity testing blood pressure monitoring is required because the CO_2_ challenge might also increase MAP. However, in clinical practice a significant modification of MAP during CVR assessment is not often seen, so that the increased MCAv is completely attributed to the vasodilatatory action of CO_2_. Yet, tests with significant increases in blood pressure should be rightly discarded as measures of the vascular diameter changes with CO_2_.

Several reports (Paulson et al. 1990; Garnham et al. 1999; Singhal and Markus 2005) have highlighted that CVR and dynamic CA (dCA) are testing different cerebrovascular control properties, but pCO2 levels influence the properties of dCA (Panerai et al. 1999). In a more recent study (Gommer et al. 2008), dCA parameters were determined during a CVR test. The results illustrate the ambiguity of the relation between CVR and dCA testing. On the one hand, there was a clear-cut effect of arteriolar diameter on dCA: increased CBF velocities due to the diameter increase in the arterioles decreased dCA performance, similarly to a rubber hose where increasing the diameter diminishes the elasticity of the hose and thereby the degree to which the diameter can be varied. On the other hand, the correlation of the results of both tests was poor, which supports the idea that at least partly different phenomena are studied.

In conclusion, for clinical purposes current CVR testing is simple and rather standardized by inducing hypercapnia and calculating the change of MCAv with respect to a prestimulus condition. Instead, for research purposes further and more extensive studies are required to unveil the complete physiological mechanisms contributing to dCA and CVR, examine the interaction between CVR and dCA, and design an efficient method to investigate CA.
